# Loss of Heterozygosity and Mutations in the RAS-ERK Pathway Genes in Tumor Cells of Various Loci in Multiple Myeloma

**DOI:** 10.3390/ijms25179426

**Published:** 2024-08-30

**Authors:** Maiia Soloveva, Maksim Solovev, Natalya Risinskaya, Elena Nikulina, Igor Yakutik, Bella Biderman, Tatiana Obukhova, Yulia Chabaeva, Sergej Kulikov, Andrey Sudarikov, Larisa Mendeleeva

**Affiliations:** National Medical Research Center for Hematology, Novy Zykovski Lane, 4a, 125167 Moscow, Russiarisinska@gmail.com (N.R.); igorya90@list.ru (I.Y.); bella_biderman@mail.ru (B.B.); uchabaeva@gmail.com (Y.C.); dusha@blood.ru (A.S.);

**Keywords:** multiple myeloma, liquid biopsy, plasmacytoma, STR profile, loss of heterozygosity, free circulating tumor DNA in plasma, *NRAS*, *KRAS*, *BRAF* genes

## Abstract

Multiple myeloma (MM) is a disease characterized by spatiotemporal heterogeneity of tumor clones. Different genetic aberrations can be observed simultaneously in tumor cells from different loci, and as the disease progresses, new subclones may appear. The role of liquid biopsy, which is based on the analysis of tumor DNA circulating in the blood plasma, continues to be explored in MM. Here, we present an analysis of the STR profiles and mutation status of the *KRAS*, *NRAS*, and *BRAF* genes, evaluated in plasma free circulating tumor DNA (ctDNA), CD138+ bone marrow cells, and plasmacytomas. The prospective single-center study included 97 patients, with a median age of 55 years. Of these, 94 had newly diagnosed symptomatic MM, and three had primary plasma cell leukemia. It should be noted that if mutations were detected only in ctDNA, “non-classical” codons were more often affected. A variety of adverse laboratory and clinical factors have been associated with the detection of rare *KRAS* or *NRAS* gene mutations in bone marrow or ctDNA, suggesting that these mutations may be factors of an unfavorable prognosis for MM. Liquid biopsy studies provide undeniable fundamental information about tumor heterogeneity and clonal evolution in MM. Moreover, we focus on using liquid biopsy to identify new high-risk factors for MM.

## 1. Introduction

Multiple myeloma (MM) is a complex disease of the blood system, with an aberrant plasma cell as its substrate. The complexity of the genetic structure of MM is reflected in the phenomenon of spatiotemporal heterogeneity of tumor clones [1,2,3]. This means that different genetic abnormalities can be observed simultaneously in tumors of different locations in the same patient. As the disease progresses, more mutations are acquired, and subclones that differ from the original clone arise. In this regard, two areas are of particular importance. Firstly, relying solely on a bone marrow sample to assess the risk of myeloma may lead to inaccurate result, as mentioned in our previous research [4]. In this regard, there is a clear need to develop a new or additional diagnostic tool for better risk assignment. This could be a liquid biopsy, for example. Secondly, the mechanisms behind chemoresistance and plasmacytoma development remain poorly understood. This underscores the need for further research into the evolution of tumor clones. Studying mutations and aberrant signaling pathways that contribute to tumor progression and drug resistance is essential for implementing a personalized approach to MM treatment [5]. Despite significant success in the treatment of MM, which became possible due to the introduction of new classes of drugs in combination with high-dose chemotherapy followed by transplantation of autologous hematopoietic stem cells (auto-HSCT), the disease remains incurable and in most cases steadily progresses [6,7,8]. If there is an appropriate target, the application of drugs that are not usually used to treat MM becomes justified. Thus, with mutations in the genes of the RAS signaling pathway (*BRAF* V600E), attempts are made to use vemurafenib; with *BCL-2* overexpression in patients with t (11;14), venetoclax is used; and in the case of JAK2 pathway activation, ruxolitinib. Other signaling pathways, such as NF-kB and PI3K/mTOR, are also being actively studied, and the search for new potential targets continues [5]. Since *KRAS/NRAS/BRAF* genes are involved in the MAPK pathway, the use of MAPK inhibitors in combination with immunomodulatory drugs in MM patients with mutations in these genes is being discussed [9].

There are few publications on the use of BRAF inhibitors in MM patients with a mutation in the *BRAF* V600E gene. In 2016, researchers from Switzerland reported a clinical observation of the successful use of a vemurafenib and cobimetinib combination in a young patient with refractory extramedullary MM with a *BRAF* V600E gene mutation [10]. In 2018, the results of a multicenter clinical trial on the effectiveness of vemurafenib in patients with different types of cancer (VE-BASKET) were presented. Nine patients with refractory MM were included in this study. In two of them, vemurafenib therapy proved to be highly effective, and the patients were in long-term remission (more than 20 months) at the end of the study. The authors suggest that the lack of antitumor effect in other patients may be explained by the fact that the *BRAF* V600E gene mutation was not the main tumorogenic event [11]. A prospective multicenter phase 2 trial evaluated the effects of combining BRAF/MEK inhibition with encorafenib and binimetinib in 12 relapsed/refractory MM patients who had a *BRAF* V600E mutation. It is important to note that the patients were heavily pre-treated. The overall response rate was high (83.3%), and the median progression-free survival was 5.6 months, indicating this therapy as a successful targeted approach [12]. Another study examined the development of resistance of tumor plasma cells to treatment with BRAF inhibitors. The authors showed that the adaptation of a tumor cell to targeted therapy is based on transcriptional state changes and epigenetic regulation [13].

Currently, in the pathogenesis of MM, not only the mutation profile of oncogenes and tumor suppressor genes are being studied, but also their interaction in the course of the disease. Oncogenic relationships between primary events (translocations and hyperdiploidy), mutations in driver genes, and areas of copy number variations have been proven. For example, associations were found between t(4;14) and mutations in the *FGFR3*, *DIS3*, and *PRKD2* genes. Two surrogate markers of DNA instability were also identified—the APOBEC mutation signature and loss of heterozygosity (LOH). A correlation was found between the degree of LOH and *TP53* gene lesions. It is expected that identifying further relationships will allow for a personalized approach to patient therapy, thereby improving its effectiveness [14].

The course of MM can vary from slowly progressing to lightning-fast forms. It depends on the degree of evolution of the tumor clone, the magnitude of the tumor load, and the presence of adverse risk factors. The signs of high-risk myeloma are diverse and include a range of laboratory and clinical parameters. The issue of searching for new prognostic factors, as well as tools for their detection, is especially relevant due to the existence of patients who, despite the absence of known high-risk factors, failed to achieve a deep antitumor response. Proteomic and genomic studies are currently being conducted to identify additional diagnostic and/or prognostic parameters of MM. Biomarkers determined noninvasively in the circulating bloodstream (microRNA and extracellular DNA) are being studied [15]. The efficacy for detecting markers missing in bone marrow while present in ctDNA for MM monitoring are being investigated [16,17,18].

The question of why in some MM patients a tumor plasma cell is able to exist outside bone marrow, forming plasmacytomas in other organs and tissues, is extremely interesting. The genetic parameters of plasmacytomas are less studied due to the lack of opportunity to analyze a large number of samples—plasmacytoma biopsy is not a mandatory procedure for MM diagnosis. The invasive nature of plasmacytoma biopsy is another incentive for the use of liquid biopsy.

Here we present a study of MM tumor clones’ heterogeneity assessed at disease onset by a wide range of methods in attempt to clarify the potential role of liquid biopsy as a diagnostic and risk stratification tool for MM. The LOH in various loci was studied by multiplex STR-PCR. The mutation status of the *KRAS*, *KRAS*, and *BRAF* genes in ctDNA, CD138+ bone marrow cells, and plasmacytomas was analyzed by AS-PCR and NGS.

## 2. Results

Table 1 shows the clinical and laboratory parameters of patients at the onset of multiple myeloma. The majority of patients (60%) were diagnosed with stage IIIA of the disease according to Durie–Salmon. Plasmacytomas were detected in 59% of patients (*n* = 57) at the onset of MM. The vast majority of patients had bone plasmacytomas (*n* = 51), in 3% of cases (*n* = 2)—extramedullary, and in 7% of cases (*n* = 4) both bone and extramedullary plasmacytomas were simultaneously detected. Cytogenetic examination by the FISH method was performed in all patients. In 46% of cases, the cytogenetic risk was determined as standard, in 51% of observations, high-risk cytogenetic aberrations were present, and in another 3% of the cases, the study could not be performed for technical reasons.

Table S1 shows the spectrum of high-risk cytogenetic aberrations detected in patients with MM. In 20.4% of cases, two high-risk anomalies were detected simultaneously in various combinations (double-hit myeloma), and three aberrations (triple-hit myeloma) were found in one observation.

**Figure 1:**
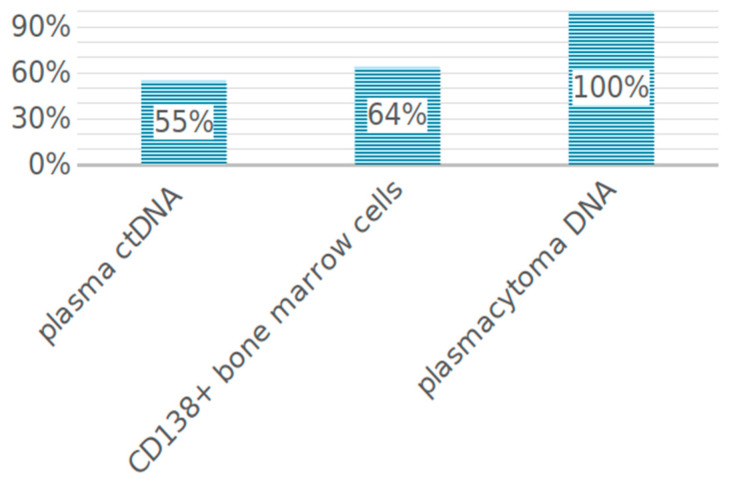
LOH frequency in tumor cells from different loci.

STR profile of paired tumor samples (ctDNA and bone marrow) of 93 patients with MM was analyzed. In nine patients, LOH was studied in three localizations: ctDNA, bone marrow, and plasmacytoma. LOH was detected in the MM substrate with different frequency depending on the tumor location. Thus, in 55% of patients (*n* = 51), aberrant STR loci with LOH were detected in ctDNA. In CD138+ bone marrow cells, the frequency of detection was higher—loci with LOH were found in 64% of patients (*n* = 60) (Figure 1).

**Figure 2:**
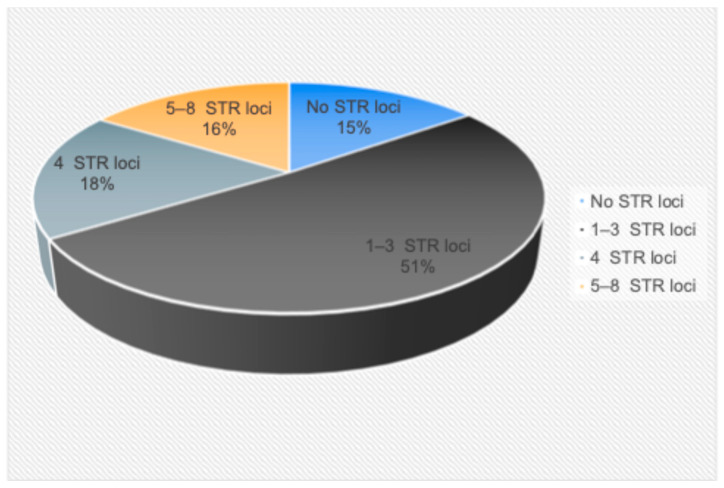
The frequency of LOH detection in ctDNA or bone marrow.

Figure 2 shows the frequency of detection of a different number of aberrant STR loci with LOH in MM. In the majority of patients (51%, *n* = 48), one to three aberrant STR loci were found in the bone marrow or ctDNA. There was no LOH in either bone marrow or ctDNA in 15% of MM patients (*n* = 14). In 18% of patients (*n* = 17), four aberrant STR loci were found in the tumor substrate. Five or more loci with LOH were identified in another 16% of patients (*n* = 15).

**Figure 3:**
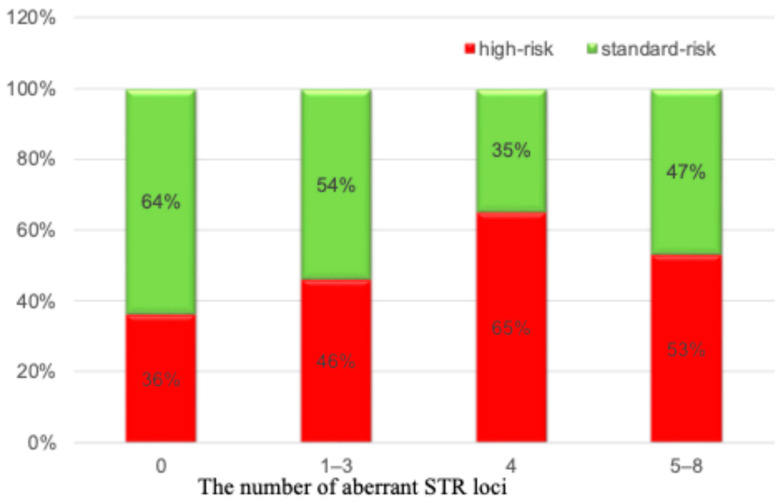
The frequency of detection of high-risk cytogenetic aberrations depending on the number of loci with LOH.

We hypothesized that patients with a large number of LOH loci would be more likely to have high-risk cytogenetic aberrations as a reflection of a large tumor load and genetic instability. We analyzed the frequency of detection of high-risk cytogenetic anomalies in each subgroup. As can be seen from Figure 3, there was no convincing evidence that with an increase in the number of loci with LOH, high-risk cytogenetic aberrations were more often observed.

There was also no association found between the number of loci with LOH and the presence of plasmacytomas. Thus, among patients who did not have LOH, 43% had plasmacytomas (6 out of 14). Among patients with one to three aberrant loci, there were 69% of cases of plasmacytomas (33 out of 48). Plasmacytomas were detected in 47% of patients (8 out of 17) with four LOH loci. Finally, among patients with a large number of aberrant loci (five to eight), 60% of cases were with plasmacytomas (9 out of 15).

The analysis of ctDNA in plasma was performed for 93 patients with MM. A concordance analysis was performed for each locus in paired tumor samples from 93 patients with MM (Figure S1). We compared the concentration of free plasma circulating DNA in patients with plasmacytomas (*n* = 55) and without them (*n* = 38). There were no differences in values: in patients with plasmacytomas, the concentration was 20 ng/mL (range 2–207), and in patients without plasmacytomas, it was 17 ng/mL (range 2–623).

The length of ctDNA fragments varied from 160 to 450 bp. The Figure S1 shows the distribution of LOH loci depending on the length of ctDNA. Mostly LOH loci were found on ctDNA fragments with a length of 400 bp. It should be noted that when analyzing ctDNA samples with fragment lengths of 160, 170, and 450 bp no LOH loci were detected.

**Figure 4:**
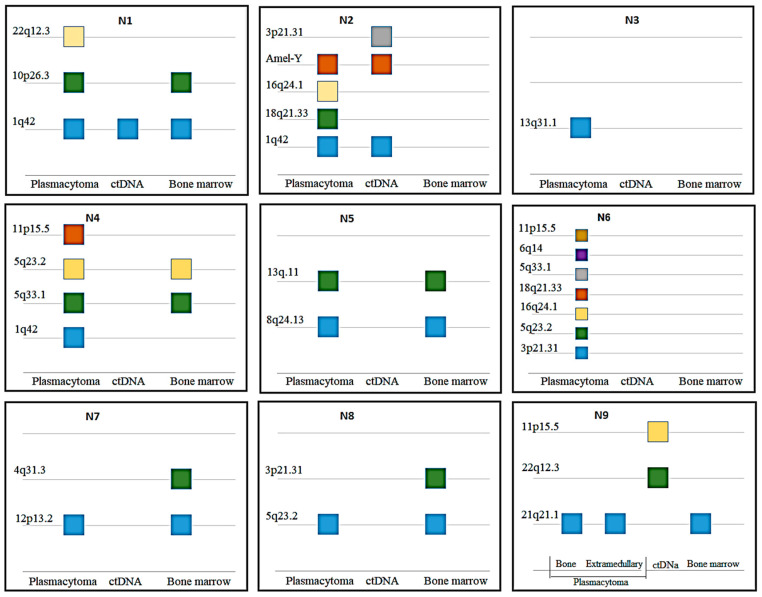
Comparison of STR loci with LOH in plasmacytomas, bone marrow, and ctDNA from nine MM patients.

For nine patients, LOH was tested using DNA from three different sources: ctDNA, bone marrow, and plasmacytomas. Additionally, in one patient, two plasmacytomas were analyzed—one from the bone and one extramedullary (Figure 4).

**Figure 5:**
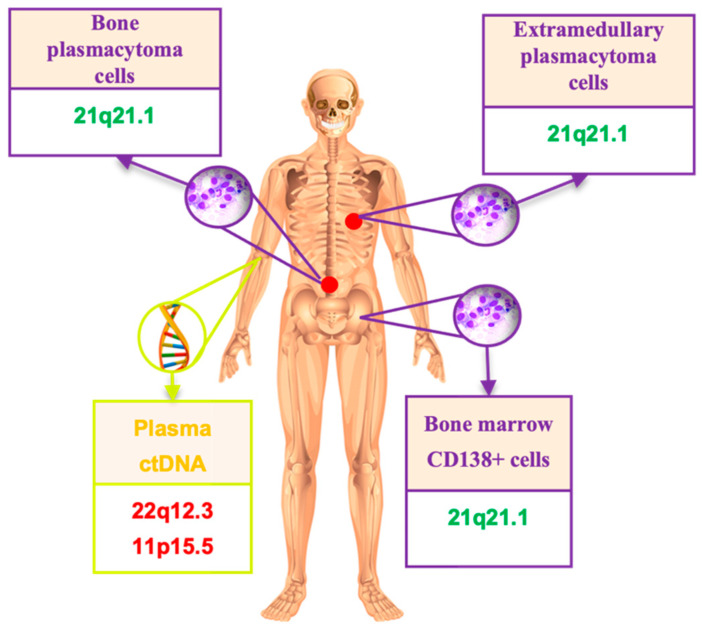
An example of LOH mismatch in DNA from different MM loci. Green highlights the same STR locus in bone marrow and two plasmacytomas, while red marks two different loci found in ctDNA.

No cases were found with the complete identity of the STR profiles in different tumor locations. Loci with LOH have always been found in plasmacytomas. Plasmacytoma profiles were more similar to that in bone marrow rather than in ctDNA (in six cases). In one interesting observation (#6), seven aberrant STR loci were identified in plasmacytoma DNA, but no LOH was found in bone marrow and ctDNA. Patient #9 with four evaluated sources had the same STR profile in bone marrow, bone and extramedullary plasmacytomas, and two other STR loci with LOH in ctDNA (Figure 5).

Somatic mutations in the *NRAS*, *KRAS*, and *BRAF* genes were evaluated for 91 patients with MM and were found in 44 of these patients (48.5%). Mutations only in the *NRAS* gene were detected in 16.5% of cases (*n* = 15), only in the *KRAS* gene—in 18.7% of cases (*n* = 17), and only in the *BRAF* gene—in 10% of cases (*n* = 9). In another 3.3% of cases (*n* = 3), two mutations in different genes were detected simultaneously: in two patients, mutations in the *BRAF* (V600E) and *NRAS* (Q61H) genes, and in one case—mutations in the *KRAS* (Q61R) and *NRAS* (Q61R) genes. We also noted two cases with two mutations of the same gene: one patient had two mutations in the *KRAS* gene (G12A, Q61L), and the second patient had two mutations in the *NRAS* gene (Q61K and Q61R).

**Figure 6:**
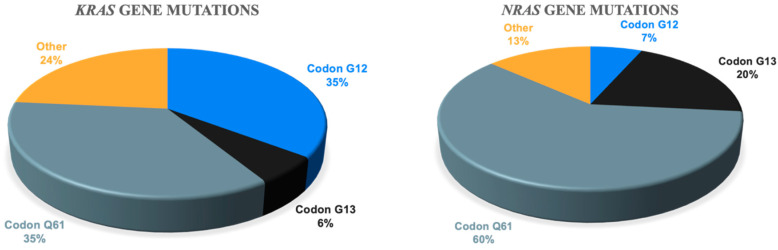
The frequency of *KRAS* and *NRAS* mutations in different codons.

Most of the *KRAS* and *NRAS* gene mutations we found affected the “classic” codons—12 and 13 for *KRAS*, and 61 for *NRAS*. However, we also found some rare variants (Figure 6).

The analysis of paired tumor samples (bone marrow and ctDNA) was performed in 39 MM patients. Mutations in the studied genes were found in 24 patients, while identical patterns in paired tumor samples were detected only in five cases (21%). Mutations of any of the three genes were found in the bone marrow of 21 patients. Mutations of one of the three genes were detected in the ctDNA of nine patients, while in five cases, the same mutations were found in bone marrow. In the remaining four observations, the mutations detected in ctDNA did not correspond to those found in bone marrow. In one case, different *KRAS* gene mutations were detected in paired tumor samples. In three other cases, *KRAS* and *NRAS* gene mutations detected in ctDNA were absent in bone marrow. Although the number of patients in each group was small, there was a trend towards more frequent detection of high-risk cytogenetic aberrations in patients with MAP kinase gene mutations in ctDNA with the absence of these mutations in the bone marrow. Further analysis in patients with RAS-ERK cascade gene mutations, which were exclusively identified in ctDNA, revealed not only that high-risk cytogenetic aberrations were more frequently detected, but also that other unfavorable prognostic factors were present. In addition, it was noted that in three out of four cases (75%), with mutations detected only in ctDNA, “non-classical” codons were affected.

Table 2 shows the data for four patients whose ctDNA had any mutations in the studied genes, as well as for three patients whose bone marrow had rare mutations. As can be seen from the table, these patients had various unfavorable prognostic factors, which reflects the concept of “high-risk myeloma”. Thus, in patient #1, with a single nucleotide deletion (c.286 delT) in the *KRAS* gene in addition to cytogenetic aberrations, high LDH activity, an advanced ISS stage, concomitant AL-amyloidosis was observed, and the course of the disease was refractory and recurrent. Despite the implementation of high-dose chemotherapy and auto-HSCT in some patients (#2, 4, and 6), complete remission was not achieved. In the other two cases with rare *NRAS* and *KRAS* gene mutations (#5 and 7), the aggressive course of the disease precluded the completion of the therapy program, and the patients died during induction treatment. These findings suggest the need for further research in this area.

In four patients, we compared the mutation profiles of the RAS-ERK cascade genes not only in bone marrow and ctDNA, but also in the plasmacytoma. In two patients, there was a mutation in the *KRAS* or *NRAS* gene in plasmacytoma tissue, while the corresponding gene was not mutated in bone marrow or ctDNA. The presence of a mutation in plasmacytoma DNA without a corresponding mutation at a different locus suggests the existence of different tumor clones in MM. Bone marrow clearance and immunochemical remission while maintaining a plasmacytoma are probably due to the clonal heterogeneity of the tumor.

Interesting data were obtained from 86 patients who underwent both STR profiling and gene mutation analysis. In patients with a larger number of LOH loci (5–8), mutations in RAS-ERK cascade genes were detected significantly more frequently compared to patients with a smaller number of LOH loci (0–4). The results showed a significant difference between the two groups (73% vs. 44%, *p* = 0.048), Table S2.

In 19 of the 39 patients with the mutation profile of RAS-ERK cascade genes analyzed in paired tumor samples, a mismatch of mutations in cDNA and bone marrow was noted. We compared the results of STR profiling in two tumor loci in this group of patients (Table 3). In the first five patients, no aberrant loci with LOH were detected in the ctDNA, and the RAS-ERK cascade genes were not mutated. One can speculate that the concentration of tumor ctDNA in these patients may have been insufficient for the detection of aberrations by one method or another. However, this assumption was not confirmed (see the last column of the table). In the remaining 14 cases, we saw not only the different status of MAP kinase genes, but also differences in the loci with LOH in the two tumor locations. Thus, in the case of nine with a mutation in the *NRAS* gene, eight aberrant loci with LOH were detected in bone marrow. At the same time, the mutation was absent in ctDNA, and seven loci with LOH were identified, of which only two were shared between ctDNA and bone marrow. The importance of studying different tumor locations to obtain a more complete picture of the MM genetic landscape is therefore evident.

## 3. Discussion

According to the relevant criteria developed by the International Myeloma Working Group (IMWG-2014), the diagnosis of the disease requires the detection of a tumor substrate (detection of more than 10% of plasma cells in bone marrow or histologically proven plasmacytoma) in combination with signs of the CRAB symptom complex and/or markers of tumor activity [19]. Therefore, one of the possible criteria for MM is a plasmacytoma, but it does not occur in all patients. To date, there is no unified classification of plasmacytomas, which causes confusion in the terminology. Thus, there are many different terms in the scientific literature: soft tissue component, extramedullary lesion, bone, intraosseous, extraosseous, extramedullary plasmacytoma. Despite the variety of definitions, we are talking about two types of plasmacytomas—anatomically related to bone and arising autonomously in various organs and tissues. Some authors refer to the term “extramedullary lesion” as anatomically disconnected from bone [20,21,22]. Other experts consider it important to differentiate these two types of plasmacytomas, calling extramedullary only lesions resulting from the hematogenous spread of a tumor cell into organs and tissues [23,24,25]. In our opinion, it is not correct to combine these two types of plasmacytomas into one concept. Bone plasmacytomas occur in about half of the patients with MM. Modern treatment strategies, high-dose chemotherapy with auto-HSCT can minimize the adverse effect of bone plasmacytomas on patient survival. Extramedullary plasmacytoma is a rarer variant of the lesion (1.7–4.5%) resulting from the spread of a tumor plasma cell outside the bone marrow by a hematogenic pathway. This is a factor of extremely unfavorable prognosis. The course of MM complicated by an extramedullary lesion is characterized, as a rule, by an unstable antitumor response or its absence, and extremely unfavorable survival rates despite the entire spectrum of modern therapy [25,26]. The pathogenesis of plasmacytoma development continues to be studied. The literature presents the results of studies of plasmacytomas on limited samples of patients [27,28,29,30]. Given the rare incidence of extramedullary lesions, it becomes obvious that multicenter collaboration is required to accumulate sufficient patient samples and implement an adequate study. LOH is a manifestation of genetic instability, characterized by the loss of one of the alleles in specific regions of the genome. It is promising to study this phenomenon in various myeloma loci. Several methods can be used to study LOH. Comparative genomic hybridization can help identify submicroscopic deletions and duplications. We previously studied the molecular karyotype of an extramedullary plasmacytoma [31]. STR profiling, on the other hand, is not a very informative method, as it only examines selected DNA markers in the genome. However, it has the advantage of being simple to perform and can be applied to the analysis of DNA samples of any quality, even those that are fragmented or have low concentrations, as is typical for ctDNA [32,33]. In this study, STR profiling was used to analyze a large number of tumor samples from different locations, and interesting information about the spatial heterogeneity of MM was obtained. Considering the limitations of this method, we conclude that it could be used for routine screening, as it is relatively inexpensive and widely available.

Liquid biopsy is considered as a non-invasive alternative to traditional tissue biopsy methods. The study of ctDNA in plasma is a promising approach for research in plasmacytoma pathogenesis. In a work published by Chinese authors, the mutation profiles of ctDNA, bone marrow, and plasmacytomas were compared. The researchers showed that the same mutations in genes were more often detected in plasmacytomas and ctDNA than in bone marrow. The authors concluded that a liquid biopsy can provide insight into the genetic structure of a plasmacytoma, and in addition, serves as a predictor of recurrence. Thus, in several patients whose ctDNA was studied in dynamics, a positive correlation was noted with blood immunochemistry data, while a paraprotein was detected later than a mutation in a certain gene [34]. The advantages of a liquid biopsy are obvious: the procedure of taking blood can be repeated multiple times and does not require a special surgical intervention of the patient, unlike a tumor biopsy. A liquid biopsy makes it possible to study the spectrum of mutations in the tumor even if a plasmacytoma biopsy is not available (for example, due to difficulties in surgical access). In this paper, we analyzed LOH in paired tumor samples. Comparing the STR profile of CD138+ bone marrow cells, plasmacytomas, and ctDNA, we noticed that the STR loci affected in plasmacytomas more often shared with those found in bone marrow than with those found in ctDNA. The question of why tumor markers are sometimes present in peripheral blood circulation in MM patients that are not found in bone marrow or in plasmacytomas seems relevant and requires further study.

In addition, we analyzed the mutation status of MAP kinase genes in bone marrow, ctDNA, and plasmacytoma. According to a recent exome-wide sequencing study, mutations in the *KRAS*, *NRAS*, and *BRAF* genes are reported in 21%, 19%, and 7% of patients with newly diagnosed MM [35]. We measured the frequency of mutations at various codons in the *KRAS* and *NRAS* genes in patients with MM. The results obtained are consistent with those presented in the literature [14]. Specifically, we found mutations affecting codon 12 of the *KRAS* gene in 35% of cases, codon 61—in another 35%, and codon 13—in 6%. According to a study published in 2018, these frequencies were 34%, 35%, and 13%, respectively. We report mutations affecting codon 61 in 60% of cases, while Walker et al. report it in 81%. The authors note that mutations in “non-classical” codons such as Q22, Y64, K117, and A146 of the *KRAS* gene appear in 18% of cases [14]. We found rare mutations not only in the *KRAS* gene, but also in the *NRAS* gene in 24% and 13% of cases, respectively. Specifically, L19F, A59G, V29A, and c.286 delT (in the *KRAS* gene), and L95P and Y64N (in the *NRAS* gene) were encountered. In all patients with rare *KRAS* or *NRAS* gene mutations, a variety of different laboratory and clinical adverse factors were noted. Additionally, if the mutation was found only in ctDNA, “non-classical” codons were affected in 75% of cases. These findings clearly require further investigation on a larger sample of patients. However, it can already be assumed that the identification of these rare mutations may be a factor of an unfavorable prognosis for MM.

Mutations in the BRAF gene are present in various types of neoplasia and cause activation of the Ras-Raf-MEK-ERK signaling pathway. Mutations in the *BRAF* gene are detected in 7% of patients with newly diagnosed myeloma [35]. Here we report a *BRAF* V600E gene mutation in 10% of cases. However, *BRAF* mutations in MM can also affect other codons. Thus, in a paper published in 2018, a unique predominance of *BRAF* D594N variants in the t(14;16) subgroup is noted [14]. Further study of *BRAF*-positive myeloma is required to understand the mechanisms of activation of the signaling pathway and determine the role of targeted drugs in treatment. Clarifying which mutation in the BRAF gene is present in the patient is of practical importance, since BRAF inhibitors are selective against the *BRAF* V600E mutation [36]. Due to a different biological mechanism of action, it is impractical to use BRAF inhibitors in patients with a *KRAS*, *NRAS*, and *BRAF* D594N mutation [37].

In 2018, the entire exome of various ctDNA and bone marrow samples was sequenced in patients with MM. The authors noted a high coincidence in the detection of clonal somatic mutations and copy number variations between ctDNA and bone marrow samples. In addition, mutations that occur exclusively at a particular locus have been identified. The researchers conclude that a joint analysis of all localizations is required to assess complete genetic profile of the tumor [38]. Our data clearly demonstrate the presence of diverse tumor clones in MM. We also observed that patients with a high number of abnormal STR loci are significantly more likely to have mutations in the RAS-ERK pathway genes compared to patients with a lower number of LOH loci (73% vs. 44%, *p* = 0.048). This suggests that with increased genetic instability of the tumor, reflected by a greater number of abnormal loci with LOH, there is also an increased level of somatic mutations in the RAS-ERK pathway genes.

The literature mentions such a phenomenon as allele drop out (allele loss) in STR loci at low concentrations of the studied DNA. However, the authors determine small amounts of DNA in the range from 25 to 100 pg of DNA per PCR sample [39]. In the cohort of patients we studied, plasma concentrations of ctDNA were high enough, which allowed us to take 1 ng of ctDNA or more into PCR. Therefore, we assume that the loss of the allele in the STR loci in ctDNA we observed is associated with aberrations in the tumor genome and its heterogeneity, and not with stochastic effects at low DNA concentrations. Our confidence is confirmed by the coincidence of aberrant STR profiles of genomic and ctDNA in some patients, as well as discrepancies in the mutation profile of *KRAS*, *NRAS*, and *BRAF* genes in ctDNA and genomic DNA from selected CD138+ cells in certain patients. According to our data, the concentration of ctDNA did not differ significantly depending on the presence or absence of plasmacytomas in patients. The median concentration of ctDNA in patients with plasmacytomas was 20 nanograms per milliliter (ng/mL), while it was 17 ng/mL for patients without plasmacytomas. These findings contradict the results reported by Chinese authors, who found higher ctDNA levels in patients with plasmacytomas than in those without [34]. However, it is important to note that the patient samples are considerably different. We included 93 patients, 55 with plasmacytomas, while Long et al. analyzed data from 18 patients, eight of whom had plasmacytomas.

The studies of ctDNA as a tool for assessing minimal residual (MRD) disease in MM are ongoing. The small length of the ctDNA fragments limits the applicability of the method. The peculiarities of ctDNA metabolism are also obstacles to its use. The detection of MRD in bone marrow along with plasma MRD negativity indicate that it is necessary to increase the sensitivity of ctDNA detection [40,41]. Further standardization of ctDNA isolation and analysis methods is required. The applicability of full-exome sequencing in routine clinical practice to determine MRD is still not obvious. However, MRD evaluation was not part of this study’s objectives. Instead, we used liquid biopsy as a diagnostic tool to identify new high-risk factors for myeloma. Thus, the value of liquid biopsy in fundamental scientific research on MM is undeniable. However, we assume that ctDNA could also be useful for applied purposes.

## 4. Materials and Methods

The prospective single-center study included 97 patients (43 men, 54 women) aged 29 to 83 years (median 55), 94 with newly diagnosed symptomatic MM, three patients with primary plasma cell leukemia. All patients attended the National Medical Research Center for Hematology (Moscow, Russia) from 21 September 2021 to 21 February 2024. The diagnosis was established in accordance with the criteria of IMWG-2014. To assess the bone system, patients underwent a low-dose CT scan of the entire body.

A positive immunomagnetic selection of CD138+ bone marrow cells was performed using a monoclonal antibody to CD138 (STEMCELL Technologies, Vancouver, BC, Canada) according to the manufacturer’s protocol. A FISH study of CD138+ cells was performed using DNA probes to detect translocations 14q32/High, 8q24/*MYC*, deletions 17p13/*TP53*, 13q14, 1p32, amplification 1q21, and multiple trisomies (Wuhan HealthCare Biotechnology, Wuhan, Hubei, China). Upon detection of t(4;14), t(14;16), del17p13, and 1q21 amplification, the patient was classified to a high cytogenetic risk group.

DNAs were isolated from samples of various localization (blood plasma, CD138+ bone marrow cells) for all patients. In addition, plasmacytoma DNA was isolated from a tumor biopsy in 9 patients. The tumor STR DNA profile was examined relative to the STR profile of the control DNA isolated from the buccal epithelium by multiplex STR-PCR (COrDIS Plus kit (Gordiz LLC, Moscow, Russia) followed by fragment analysis as it was described previously [4]. For all patients with a change in the allele balance in heterozygous STR loci of tumor DNA compared to the control one, a relative decrease in the level of the fluorescent signal of the minor allele (in percent) was calculated.

The mutational status of the *KRAS*, *NRAS*, and *BRAF* genes was studied in 91 patients. In all patients, the study was conducted on genomic DNA isolated from bone marrow samples; in 39 patients, plasma ctDNA samples were additionally examined; and in 4 patients, DNA from three localizations (plasma ctDNA, bone marrow, plasmacytoma) was used. Isolation of genomic DNA from bone marrow samples and sections of paraffin embedded plasmacytoma biopsies was performed according the methodologies described earlier [42,43]. CtDNA from blood plasma was isolated with a QIAamp MinElute ccfDNA Mini Kit (QIAGEN, Hilden, Germany). PCR amplification was performed with a C1000 TouchTM PCR thermal cycler (“Bio-Rad”, Hercules, CA, USA) using oligonucleotide pairs specific for exons 2 and 3 of *KRAS* (KRAS-E2F: 5′-CTTAAGCGTCGATGGAGGAG-3′; KRAS-E2R: 5′-GAATGGTCCTGCACCAGTAA-3′; KRAS-E3F: 5′-ATAATCCAGACTGTGTTTCTCCC-3′ and KRAS-E3R: 5′-AAAACAGGGATATTACCTACCTCAT-3′) and NRAS (NRAS-E2F: 5′-GCTCGCCAATTAACCCTGA-3′; NRAS-E2R: 5′-ACAGGTTTTAGAAACTTCAGCAG-3′; NRAS-E3F: 5′-AGGGACAAACCAGATAGGCA-3′ and NRAS-E3R: 5′-ACCTCATTTCCCCATAAAGATTCA-3′) genes. Each PCR reaction was carried out in a volume of 25 µL containing 2.5 µL of (×10) a PCR buffer (Syntol, Moscow, Russia), 0.25 mM of a dNTPs solution (Syntol, Russia), 0.25 mM of MgCl_2_ solution (Syntol, Russia), 5 pmol of each primer, 200 ng of genomic DNA, and 1E of SynTaq DNA-polymerase (Syntol, Russia). PCR was carried for 40 cycles with 30 s ofdenaturation at 92 °C and 20 s of annealing at 57 °C followed by a 30 s extension at 72 °C. Cycling was started by 2 min of denaturation at 92 °C and terminated by 10 min of incubation at 72 °C.

Mutations in *KRAS* and *NRAS* genes were identified by high-throughput sequencing (MiSeq, Illumina, San Diego, CA, USA), with confirmation of the findings by Sanger sequencing (Nanophor 05, Institute of Analytical Instrumentation of RAS, Moscow, Russia).

Sequencing libraries were prepared from the accumulated amplicons using the «Nextera XT DNA Library Prep» and «Nextera XT Index Kit v2» (Illumina, USA) according to the manufacturers recommendations. Sequencing was performed with a MiSeq genetic analyzer (Illumina, USA) using the «MiSeq Reagent Micro Kit v2 300-cycles» («Illumina», USA). Data filtration, service sequence deletion, mapping of readings, and search and annotation of variants was carried out with the Trimmomatic [44], BWA [45], SAMtools [46], Vardict [47], and Annovar [48] utilities. The information about clinical significance of the mutations was obtained from open sources (COSMIC and DB SNP).

Sanger sequencing was carried out with a BigDye Terminator v1.1 Cycle Sequencing Kit (Applied Biosystems, Waltham, MA, USA) using a Nanophor 05 genetic analyzer (Institute of Analytical Instrumentation of RAS, Russia).

The *BRAF* V600E mutation was determined by real-time allele-specific PCR (CFX96 Touch, Bio-Rad, USA) according to the methodology described earlier [49].

We used standard methods of descriptive statistics and frequency analysis. To test hypotheses about differences in the distributions of categorical characteristics in comparison groups, we used contingency table analysis and two-sided Fisher’s test to assess the level significance. To assess the level of agreement between studies, we used AC1 (Gwet’s first-order agreement coefficient).

## 5. Conclusions

Spatial heterogeneity of tumor clones in MM were assessed using a variety of approaches. Genetic abnormalities in DNA isolated from various sources, including plasma (ctDNA), CD138+ bone marrow cells, and plasmacytomas, were compared. The study of the STR profile in DNA isolated from different tumor localizations demonstrated the anatomical heterogeneity of MM. Future studies are needed to clarify whether the number of STR loci with LOH has prognostic value in MM. The detection of rare mutations in the *KRAS* and *NRAS* genes in MM is associated with a variety of clinical and laboratory adverse factors. Relying solely on a bone marrow sample for risk stratification in MM may lead to an inaccurate result. Given the detection of aberrations in tumor ctDNA that differ from those in bone marrow and plasmacytoma, the issue of practical use of liquid biopsy for risk stratification is being discussed.

## Figures and Tables

**Table 1:** General characteristics of the patient sample.

Parameters	Patients with MM (*n* = 97)
Age, years, median and range	55 (29–83)
Males/females	43/54
Type of secretion	
G	59 (61%)
A	19 (20%)
BJ	15 (15%)
D	4 (4%)
Type of FLC	
κ	58 (60%)
λ	39 (40%)
D-S stage	
IA	5 (5%)
IB	2 (2%)
IIA	16 (17%)
IIIA	58 (60%)
IIIB	15 (15%)
Not available	1 (1%)
ISS stage	
I	29 (30%)
II	20 (21%)
III	17(17%)
Not available	31 (32%)
Hemoglobin (g/L), median and range	109 (66–156)
LDH (U/L), median and range	170 (65–694)
% plasma cells in bone marrow aspiration, median and range	16 (2.4–92)
FISH	
Standard risk	45 (46%)
High risk	49 (51%)
Not available	3 (3%)
Plasmacytomas	
Yes	57 (59%)
No	40 (41%)
	*n* = 57
bone	51 (90%)
extramedullary	2 (3%)
bone and extramedullary	4 (7%)

**Table 2:** Adverse prognostic factors in MM patients with rare mutation variants in the *KRAS* and *NRAS* genes.

#	Mutations in CD138+ Bone Marrow Cells	Mutations in ctDNA	Disease Features
1	none	*KRAS* c.286 delT	High LDH; ISS stage III; double-HIT: del17p13, 1q21; amyloidosis; Relapse/refractory
2	none	*NRAS* L95P	1q21; only partial remission after HSCT
3	none	*KRAS* G12V	High LDH; 1q21; relapse/refractory
4	*KRAS G12S*	*KRAS* *V29A*	T (4;14); only partial remission after 2 rounds of HSCT
5	*NRAS* Y64N	none	Plasma cell leukemia; high LDH; 1q21; extramedullary plasmacytomas; D-myeloma; death in induction
6	*KRAS* A59G	none	only partial remission after HSCT
7	*KRAS* L19F	none	ISS stage III; double HIT: t(14;16), 1q21; relapse/refractory; death in induction

**Table 3:** LOH in 19 patients with mismatch in MAP kinase gene mutations in paired tumor samples.

#	LOH Loci in Bone Marrow	Mutations in Bone Marrow	LOH Loci in ctDNA	Mutations in ctDNA	DNA Concentration (ng/mL)
1	2	*KRAS*	0	none	17.5
2	2	*BRAF*	0	none	11
3	6	2 *KRAS*	0	none	22
4	0	*NRAS*	0	none	13.5
5	2	*KRAS*	0	none	17
6	0	-	3	*KRAS*	45.75
7	3	*KRAS*	4 (shared 1)	none	46.5
8	0	*KRAS*	6	none	32
9	8	*NRAS*	7 (shared 2)	none	8
10	0	*KRAS*	1	none	10
11	0	*NRAS*	1	none	17
12	2	*KRAS*	5 (shared 1)	none	20
13	2	-	3 (shared 1)	*NRAS*	77
14	5	*KRAS G12S*	3 (shared 2)	*KRAS* V29A	17
15	2	*BRAF*	4 (shared none)	none	30
16	6	*NRAS BRAF*	3 (shared 2)	none	17
17	0	-	4	*KRAS*	50
18	5	*NRAS*	2 (shared none)	none	5
19	1	*BRAF*	1 (shared none)	none	10

## Data Availability

The data are contained within the article and Supplementary Materials.

## Supplementary Materials

The following supporting information can be downloaded at: https://www.mdpi.com/article/10.3390/ijms25179426/s1.
