# Supplementary material for: Loss of Heterozygosity and Mutations in the RAS-ERK Pathway Genes in Tumor Cells of Various Loci in Multiple Myeloma
Source: Int J Mol Sci. 2024 Aug 30;25(17):9426. doi: 10.3390/ijms25179426 (PMC11394882; doi:10.3390/ijms25179426)
Supplement: Supplementary file 1 [file ijms-25-09426-s001.zip › figS1,S2, Table S1,S2.pdf]

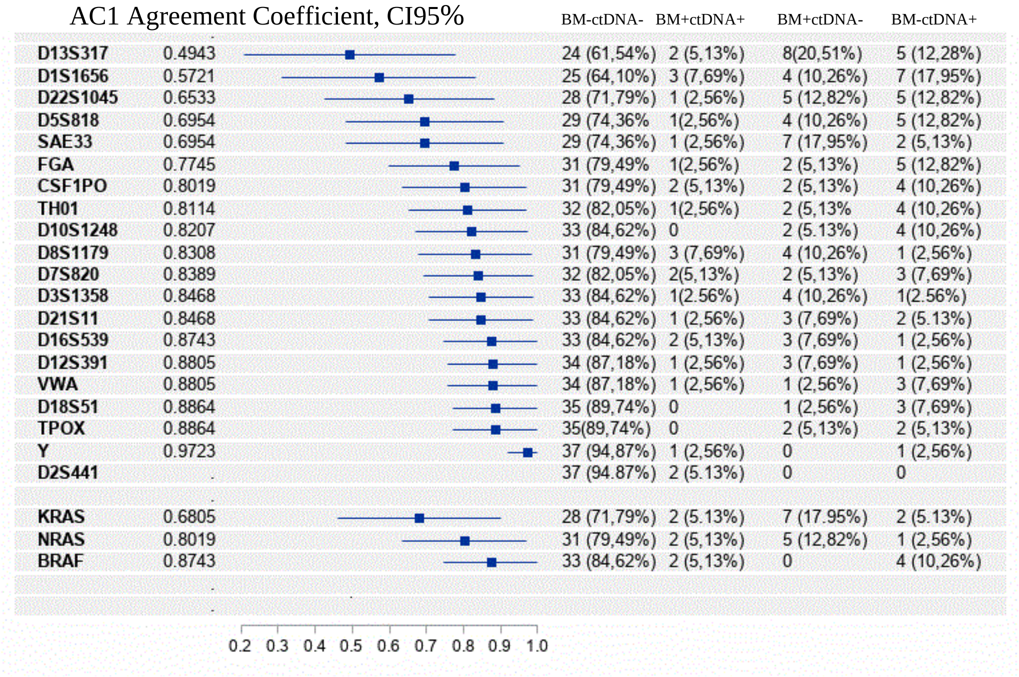


**Figure S1.** Concordance score and number of matching and discordant results for each locus and gene

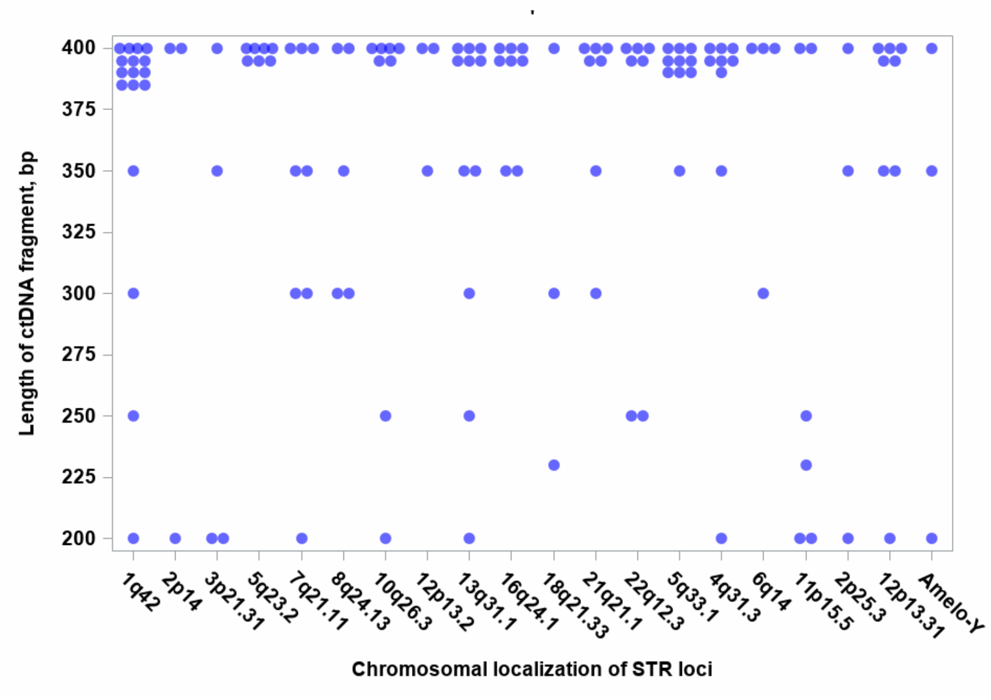


**Figure S2.** Distribution of LOH loci depending on the length of the ctDNA fragment

**Table S1.** Spectrum of high-risk aberrations in MM patients

| Cytogenetic abnormality | Patients with MM (n=49) |
|---|---|
| t(4;14) | 6 (12,3%) |
| t(14;16) | 1 (2%) |
| Gain 1q | 25 (51%) |
| del17p13 | 6 (12,3%) |
| Double-hit | 10 (20,4%) |
| t(4;14) and gain 1q | 2 |
| t(4;14) and del17p13 | 2 |
| t(14;16) and gain 1q | 2 |
| t(14;16) and del17p13 | 2 |
| del17p13 and gain 1q | 2 |
| Triple-hit del17p13, t(14;16) and gain 1q | 1 (2%) |

**TableS2.** Comparison of the mutation status of RAS-ERK cascade genes and LOH in MM

| | 0-4 LOH loci (n=71) | 5-8 LOH loci (n=15) | p |
|---|---|---|---|
| Mutations in RAS-ERK genes | 31(44%) | 11 (73%) | 0.048 |
